# Extractables and Leachables in Pharmaceutical Products: Potential Adverse Effects and Toxicological Risk Assessment

**DOI:** 10.3390/toxics14010092

**Published:** 2026-01-20

**Authors:** Samo Kuzmič, Tjaša Zlobec, Marija Sollner Dolenc, Robert Roškar, Tina Trdan Lušin

**Affiliations:** 1 Lek Pharmaceuticals d.d., Sandoz Development Center Slovenia, Verovškova 57, SI-1526 Ljubljana, Slovenia; samo.kuzmic@sandoz.com (S.K.); tjasa.zlobec@sandoz.com (T.Z.); 2 Faculty of Pharmacy, University of Ljubljana, SI-1000 Ljubljana, Slovenia; marija.sollner@ffa.uni-lj.si (M.S.D.); robert.roskar@ffa.uni-lj.si (R.R.)

**Keywords:** leachables, extractables, toxicological risk assessment, ICH Q3E, toxicological evaluation

## Abstract

During production, storage, and administration, drug products (and their intermediates) are in contact with many different types of materials, which include manufacturing components, container closure systems, and administration materials; therefore, there is a potential for their interactions and the introduction of leachables. The presence of leachables may impact key quality attributes of drug products in many ways. These include potential alterations in drug product stability, resulting in a reduced shelf-life, compromised drug product efficacy due to degradation or inactivation of active pharmaceutical ingredients, and impaired drug product physical acceptability due to precipitation, discolouration and/or change in odour or flavour. Moreover, some leachables may be inherently toxic (mutagenic, carcinogenic, immunogenic, etc.) posing direct risks to patient safety. Comprehensive toxicological evaluation of extractables and leachables is therefore essential. Documented cases demonstrate that presence of leachables can lead to serious and clinically significant adverse effects, underscoring the importance of their identification, quantification, and toxicological assessment during pharmaceutical development. This paper provides an overview of the toxicological limits used in the analyses of extractables and leachables and illustrates how they are translated into analytical limits. It also outlines the workflow for toxicological risk assessment of extractables and/or leachables, including evaluations of mutagenicity and other relevant toxicological endpoints. Special attention is given to the interpretation of the draft ICH Q3E guideline, which represents a pivotal development in harmonizing global expectations for extractables and leachables safety assessments. Understanding and correctly applying ICH Q3E is crucial, as it will shape regulatory strategies, analytical approaches, and risk management practices across the pharmaceutical industry. The paper concludes by highlighting emerging challenges that demand sustained advancements in both scientific methodologies and regulatory frameworks.

## 1. Introduction

The control of impurities is a critical and continuous challenge in ensuring the safety, quality, and efficacy of pharmaceutical products. While traditional regulatory and scientific frameworks have focused on process-related impurities from drug substance (DS) production and degradation products in the drug product (DP), a distinct class of contaminants has gained significant attention in recent decades: extractables and leachables (E&L).

**Figure 1:**
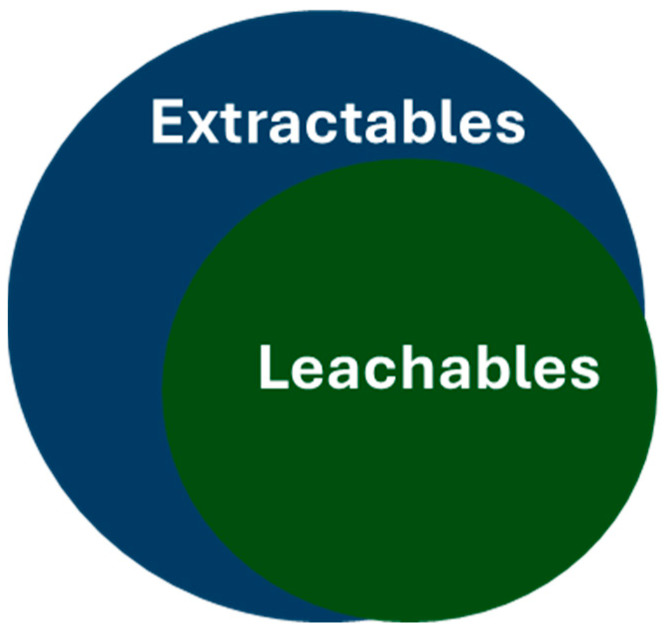
A Venn diagram illustrating that leachables are frequently a subset of extractables; however, not all leachables are necessarily extractables (adapted from [4]).

Drug products (and their intermediates) are in contact with many different types of materials during their production, storage, and administration, including manufacturing components, container closure (packaging) system, and administration materials. Therefore, there is a potential for their interactions and the introduction of leachables. Leachables are organic and inorganic chemical entities that are present in a drug product because they have leached into it from manufacturing components, packaging systems, and/or delivery systems under normal conditions of storage and use or during accelerated drug product stability studies [1,2,3]. Conversely, extractables are organic and inorganic chemical entities that can be released from manufacturing components, packaging systems, and/or delivery systems into an extraction solvent under exaggerated laboratory conditions [1,2,3]. In ideal scenarios, leachables represent a subset of extractables; however, in practice, we may encounter leachables that were not initially detected during the extractables testing (please refer to Figure 1). Potential explanations for this include the formation of secondary leachables via reactions between primary leachables and DP components, an inadequate extractable study design, the degradation of leachables into new compounds, or the introduction of new leachables resulting from material aging (e.g., due to exposure to ultraviolet (UV) light, heat, or oxygen) during long-term storage.

The presence of leachables can impact key quality attributes of drug products in many different ways. These include potential alterations to drug product stability resulting in a reduced shelf-life, compromised drug product efficacy due to degradation or inactivation of DSs, and impaired DP physical acceptability due to precipitation, discolouration, and change in odour or flavour. Furthermore, leachables may be inherently toxic (e.g., mutagenic, carcinogenic, or immunogenic) and thus pose a direct risk to patient safety. Moreover, interactions between leachables and DPs may generate new chemical entities, which may impact drug product safety; for example, DS could react with a leached substance and form a new chemical entity with immunogenic properties [5]. Consequently, the comprehensive characterization and assessment of E&L is important to ensure the quality, safety and efficacy of drug products.

## 2. Safety Risks Associated with Leachables

The universe of E&L is vast, diverse and continuously expanding; consequently, the toxicological profiles of leachables also display significant variability. Leachables generally present toxicological risks through two primary mechanisms—direct toxicity, in which the leachable substance is intrinsically toxic, and indirect, product-mediated toxicity, which results from adverse interactions between the leachable and the drug product [6]. This section reviews several classes of leachables that have attracted considerable recent attention due to their toxicological characteristics or their potential to compromise DP integrity (an overview is provided in Table 1).

Phthalates are a class of compounds used as plasticizers to impart flexibility and durability to plastic products. In medical applications, their primary source is plasticized polyvinyl chloride (PVC), which can contain up to 50% phthalates by weight, with di(2-ethylhexyl) phthalate (DEHP) being the most frequently used [7]. The toxicology of phthalates is extensively studied, with established links to adverse developmental, reproductive, and endocrine health effects [7,8,9,10]. Similarly, bisphenol A (BPA), a common leachable from polycarbonate materials, has also been associated with adverse endocrine health effects [11].

Furthermore, certain leachable classes, such as polycyclic aromatic hydrocarbons (PAHs), often associated with carbon black; 2-mercapto-benzothiazole (MBT), a vulcanization accelerator used in elastomer production; and *N*-nitrosamines, are classified as potential or known carcinogens [10]. *N*-nitrosamines, in particular, have been under intense scrutiny within the pharmaceutical industry in recent years. Leaching from manufacturing, packaging, or administration components has been identified as one of the potential root causes for their presence [12,13]. From an E&L standpoint, concerns regarding *N*-nitrosamines were first linked to elastomers, arising from the possible use of secondary amines in curing systems or their generation from vulcanization accelerators such as thiurams and dithiocarbamates during rubber compounding [10,14]. However, more recently, *N*-nitrosamine formation has been observed in primary packaging of finished products in blisters with lidding foil containing nitrocellulose [12,15]. During the blister heat-sealing process, nitrogen oxides generated from nitrocellulose can react with low-molecular-weight amines (present in printing inks or the drug product itself). This reaction results in the formation of *N*-nitrosamines, which can then transfer to the product via evaporation and condensation [12]. Most recently, in August 2025, the U.S. Food and Drug Administration (FDA) announced an investigation into the potential for *N*-nitrosodibutylamine (NDBA) and other small-molecule nitrosamine impurities to leach (migrate) from printed overwraps or pouches into drug products packaged in infusion bags [16].

Finally, metal ions represent another important class of potential leachables, exhibiting a wide range of toxicological properties that necessitate careful evaluation [17].

Beyond the inherent toxicity described above, an important mechanism of leachable toxicity is indirect, product-mediated toxicity. In these cases, leachables are not intrinsically toxic; instead, their adverse effects result from interactions with the drug product. This risk is particularly significant for biologics (e.g., monoclonal antibodies and therapeutic proteins) [6]. Unlike small-molecule drugs, biologics are large, thermodynamically less stable molecules that depend on a precise three-dimensional conformation. Their structural complexity, high molecular weight, and extensive surface area make them more vulnerable to interactions with leachables. While product quality changes, such as unfolding, oxidation, aggregation, truncation, or increased particulate matter, may not immediately raise toxicological concerns, they can nonetheless compromise patient safety, for example by triggering an immunogenic response to a modified protein [6]. Some of the adverse effects resulting from these interactions are presented below.

One of the most known cases highlighting the clinical relevance of leachables involves EPREX^®^ (epoetin alfa), where leaching of vulcanizing agents from uncoated stoppers used in pre-filled syringes has been associated with adverse clinical outcomes [18,19]. Following a reformulation that replaced human serum albumin with polysorbate 80 as a stabilizer, an increased incidence of antibody-mediated pure red cell aplasia (PRCA) was observed in patients with chronic renal failure. PRCA was attributed to the development of neutralizing antibodies against both recombinant and endogenous erythropoietin. Investigations identified leachables as a potential root cause, with studies demonstrating that polysorbate 80 facilitated the extraction of low levels of dialkylphenol disulfide (a vulcanizing agent) and its related substances from the uncoated rubber components, which had an adjuvant effect. The issue was resolved by replacing the uncoated rubber parts with fluoropolymer-coated components, which acted as an effective barrier for migration and significantly reduced leachable levels [18,19].

Tungsten has been extensively reported as leachable from pre-filled syringes, adversely affecting the quality of biological DPs [5,20,21]. Glass syringe barrels are often manufactured using tungsten pins to form the inner needle channel. Under high temperature, tungsten can oxidize in the presence of air and interact with the glass, resulting in the formation of residual tungsten species. These residues may persist despite subsequent washing steps and can come into direct contact with the drug product. Even trace levels of tungsten (of the order of 1 part per million (ppm)) have been shown to induce protein oxidation and aggregation, posing a significant risk to product quality and patient safety [5]. Seidl et al. reported tungsten-induced denaturation and aggregation of epoetin alfa, suggesting this phenomenon as a potential root cause for the increased immunogenicity observed during pre-marketing clinical trials [21]. Similar risks may arise from other metals as well. For instance, researchers at Novo Nordisk demonstrated that aluminium and zinc can adversely affect the physical stability and secondary structure of a glucagon-like peptide-1 derivative, whereas potassium and magnesium showed no such effect [22].

Phosphate-buffered formulations are susceptible to precipitation of phosphate salts when divalent cations such as Ca^2+^ or Zn^2+^ leach from elastomeric closures. Similarly, leaching of residual aluminium oxide from glass vials has been reported to induce aluminium phosphate precipitation within phosphate-buffered drug products [5].

Researchers at Amgen identified acrylic acid as a potential leachable originating from the acrylic adhesive used to affix the needle to the glass barrel of pre-filled syringes. Acrylic acid was found to be extractable at concentrations approaching 5 μg/mL under certain conditions [23]. Importantly, this compound was shown to react with therapeutic proteins via Michael addition at three distinct nucleophilic sites: the *ε*-amino group of lysine residues, the *N*-terminal amino group, and the imidazole side chain of histidine. These covalent modifications can alter the physicochemical properties of the protein, including its net charge, by converting positively charged residues into negatively charged adducts, and its hydrophobicity. Such structural alterations may compromise the stability, efficacy, and/or safety of the therapeutic protein [23].

Baderdin et al. demonstrated that formaldehyde and acetaldehyde, identified as polymer-borne leachables, interact with lysine residues of human-derived coagulation factor IX in a concentration-dependent manner. This interaction resulted in a measurable reduction in the clotting activity of the therapeutic protein. Structural analysis revealed that formaldehyde specifically reacted with lysine residues located at two distinct positions within the protein [24].

Leachables from rubber stoppers represent another well-documented cause of instability in protein-based biopharmaceuticals. These compounds have been shown to promote the formation of both soluble and insoluble high molecular weight aggregates in model proteins, such as immunoglobulin G (IgG) and erythropoietin (EPO) [25].

Adverse effects of leachables are not restricted to the container closure systems; they can also originate from other sources, such as manufacturing components and laboratory equipment. A notable example involves *bis*(2,4-di-*tert*-butylphenyl)phosphate (bDtBPP), a degradation product of the antioxidant *tris*(2,4-di-*tert*-butylphenyl)phosphite (commercially known as Irgafos^®^ 168), which is widely utilized in polyethylene formulations. Hammond et al. demonstrated that bDtBPP acts as a potent inhibitor of cell growth, leading to significant reductions in drug substance yields. In mammalian cell culture studies, bDtBPP exhibited cytotoxicity at sub-ppm concentrations. Cellular response was characterized by a rapid onset and a marked decrease in mitochondrial membrane potential. Further characterization revealed that the migration of bDtBPP from polyethylene films is dependent on both time and temperature. Notably, the generation of significant quantities of this leachable was attributed to the exposure of oxidized Irgafos^®^ 168 to ionizing radiation, such as gamma irradiation, commonly used for sterilization [26,27]. Similarly, 3,5-dinitro-bisphenol A, a leachable formed through the combination of polycarbonate moulding processes and γ-sterilization, was found to inhibit the growth of Chinese Hamster Ovary (CHO) cells when released from Erlenmeyer shaker flasks, thereby interfering with biological assay outcomes [28]. In addition, extractables from sterilizing-grade filters used during biopharmaceutical manufacturing have been identified as destabilizing agents, contributing to increased levels of protein aggregation, oxidation, and the formation of acidic species [29].

Indirect, product-mediated effects of leachables are most effectively identified through comprehensive drug product stability, compatibility and/or characterization programs [6].

**Table 1:** Overview of safety risks associated with leachables.

Leachable/Leachable Class	Toxicological/Quality Risk	Possible Sources
Phthalates (e.g., DEHP)	Reproductive and developmental toxicity; endocrine disruption [7,8,9,10]	Plasticized PVC (IV bags, tubing, catheters, etc.) [7,8,9,10]
Bisphenols (e.g., BPA)	Endocrine disruption; reproductive toxicity; potential metabolic/immune effects [11,30]	Polycarbonate plastics; Epoxy resins [11,30]
*N*-Nitrosamines (e.g., NDMA, NDBA)	Potential or known mutagenic carcinogens [10,31]	Elastomers [10,14]; reaction between nitrogen oxides from nitrocellulose lidding foil and low-molecular-weight amines [12]; printed overwraps or pouches [16]
PAHs (e.g., Benzo[a]pyrene)	Potential or known mutagenic carcinogens [10,31]	Carbon black (colorant or reinforcing agent in polymers) [10]
2-Mercaptobenzothiazole (MBT)	Probable carcinogen (IARC Group 2A) [32]	Elastomers (vulcanization accelerator) [10]
Dialkylphenol disulfide and related substances	Indirect (Product-Mediated): Adjuvant effect causing immunogenicity (e.g., PRCA in EPREX case) [18,19]	Uncoated rubber components (e.g., stoppers and plungers) [18,19]
Metals (e.g., Tungsten, Aluminium, Zinc, Calcium)	Direct: Wide range of toxicological properties depending on the specific elemental impurity [17] Indirect (Product-Mediated): Protein oxidation and aggregation (immunogenicity risk), precipitation of phosphate salts in phosphate-buffered formulations [5,20,21,22]	Glass, metal, elastomer and plastic components; Glass forming pins (tungsten) [5]
Acrylic acid	Indirect (Product-Mediated): Covalent modification of proteins (Michael addition) at Lys/His/N-term that alters charge/hydrophobicity which can compromise stability, efficacy and/or safety of proteins [23]	Acrylic adhesives (e.g., used in pre-filled syringes for needle attachment) [23]
Aldehydes (e.g., Formaldehyde, Acetaldehyde)	Direct: Potential or known mutagenic carcinogens and sensitizers [33,34] Indirect (Product-Mediated): Schiff base formation (loss of activity), which can also lead to protein cross-linking (dimerization) [24]	Various polymers—typically originating from polymer degradation, which may occur during manufacturing, sterilization, and/or due to material aging during long-term storage [24]

Abbreviations: Bisphenol A (BPA); Di(2-ethylhexyl) phthalate (DEHP); International Agency for Research on Cancer (IARC); 2-Mercaptobenzothiazole (MBT); *N*-Nitrosodimethylamine (NDMA); *N*-Nitrosodibutylamine (NDBA); Polycyclic aromatic hydrocarbons (PAHs); Polyvinyl chloride (PVC); Pure red cell aplasia (PRCA).

## 3. Toxicological Risk Assessment of Extractables and Leachables

The management of leachables is of high importance to pharmaceutical and biotechnology drug product manufacturers as well as regulatory authorities, due to the potential safety and/or quality risks posed by certain leachables when present above specific concentrations in the DPs. In the 1980s, the FDA began to formally address the issue of leachables in drug products, prompted by reports of patient sensitivities induced by leachables and other potential safety concerns associated with these substances [3,14,35,36]. Since then, the control of both E&L has become an integral part of pharmaceutical development and regulatory submissions for many dosage forms, particularly for those considered to have a high risk for packaging component-dosage form interactions, along with the high degree of concern associated with their route of administration (see Table 2) [3,36]. Routes that bypass the body’s natural defence mechanisms, or deliver substances to particularly sensitive tissues, are classified as highest/high risk and are subject to the most stringent E&L evaluation.

The potential adverse effects of a leachable on patients’ health will depend on its intrinsic toxicity and the extent of exposure, which is influenced by its concentration in the drug product as well as the drug product’s maximum daily dose, clinical dosing regimen (frequency of dosing and treatment duration), and route of administration.

### 3.1. Toxicological Limits Applicable in the Analyses of Extractables and Leachables

In E&L analyses, depending on the desired outcome of the study, two different analytical approaches can be used—screening methods or targeted methods. The purpose of a screening method is to detect, identify, and semi-quantitatively estimate the concentrations of all relevant but unspecified analytes in a test sample that exceed a predefined reporting threshold, such as the Analytical Evaluation Threshold (AET). Conversely, a targeted method aims to quantify target analytes in a defined test sample with an appropriately high degree of accuracy and precision across a specified concentration range [37]. There is also a possibility to use a combination of both approaches, namely targeted screening methods. Regardless of the chosen approach, the analytical method must demonstrate sufficient sensitivity. For non-targeted screening, the limit of detection (LOD) and limit of quantitation (LOQ) must be at or below the AET, while for targeted analyses, the LOD/LOQ must be at or below the compound-specific acceptance limit [5]. Regardless of the analytical approach, it is important to establish appropriate analytical limits, above which the E&L should be identified and (semi)quantified. These limits should be established based on appropriate toxicological thresholds.

Although leachables represent a specific class of drug product impurities, current regulatory guidance for drug product impurities (ICH Q3B) considers them outside its scope [38]. Thresholds proposed specifically for drug product leachables are typically based on either patient safety considerations or current capabilities of analytical technology. State-of-the-art analytical technology enables the detection of trace organic and inorganic compounds at extremely low concentrations (i.e., ng/mL or ng/g). However, the identification and toxicological qualification of every individual chemical entity at this level is neither necessary from a toxicological point of view nor practical. Such an approach would often require the identification and quantification of a large number of potential leachables, many of which may pose negligible toxicological risk. To address this, the concept of safety thresholds was introduced, enabling a scientific and risk-based determination of acceptable levels of leachables. These thresholds can be based on established toxicological data and additional safety risk factors that consider, for example, treatment duration, daily exposure, and route of administration [3,39,40].

In 2006, a team of toxicologists, working as part of the Product Quality Research Institute (PQRI) Leachables and Extractables Working Group, established the terms Safety Concern Threshold (SCT) and Qualification Threshold (QT) for the Orally Inhaled and Nasal Drug Products (OINDP) (Table 3) [39]. SCT was defined as the threshold below which a leachable would have a dose so low that it would represent negligible safety concerns from carcinogenic and non-carcinogenic toxic effects point of view [39,41]. While QT was defined as the threshold below which a given non-carcinogenic leachable is not considered for toxicological assessment (safety qualification), unless structure–activity relationship (SAR) concerns are identified [39,41]. Although these definitions were originally developed for leachables, the threshold concept is also applicable to extractables, as extractables studies are used to predict the leachable profile. Below the SCT, identification of leachables is generally not required. Similarly, below the QT, leachables without SAR concerns (e.g., for mutagenicity, irritation and sensitization), typically do not require compound-specific toxicological assessment [39,41]. The proposed SCT for OINDP by the PQRI is 0.15 μg/day, which is often considered an overly conservative limit [39]. For instance, the ICH M7 guideline, finalized in 2017, proposed the Threshold of Toxicological Concern (TTC) of 1.5 μg/day for potentially deoxyribonucleic acid (DNA) reactive (mutagenic) impurities without carcinogenicity data (excluding the cohort of concern group of compounds) applicable to all routes of administration [31]. The TTC is derived by linear extrapolation from the dose associated with a 50% tumour incidence in the most sensitive species and at the most sensitive site in carcinogenicity studies, down to a theoretical cancer risk of 1 in 10^6^ (corresponding to 0.15 μg/day) [31]. For pharmaceuticals, a theoretical 1 in 10^5^ cancer incidence is considered justified, resulting in a TTC value of 1.5 μg/day [31]. The TTC limit of 1.5 μg/day is based on lifetime exposures and adjustments/increase of limits for less-than-lifetime (LTL) exposures are possible as per the ICH M7 guideline [31]. The difference in the proposed limits between the PQRI for OINDP and ICH M7 guideline is due to different risk factor considerations. PQRI SCT incorporates a 10^−6^ (1 in 1,000,000) risk factor, while the ICH M7 limit of 1.5 μg/day corresponds to a 10^−5^ (1 in 100,000) risk factor. A lower threshold for leachables in OINDP was proposed by the PQRI, reflecting the direct delivery of some of these dosage forms to diseased organs of a sensitive patient population. This conservative approach was further justified by the increased likelihood of detecting chemicals of concern as leachables, particularly in metered dose inhalers [14,39,41]. For OINDP, certain “special case” compounds and compound classes were identified by PQRI as those requiring lower safety thresholds due to specific toxicological concerns. These include PAHs (sometimes referred to as polynuclear aromatic hydrocarbons (PNAs)), *N*-nitrosamines, and the individual chemical entity 2-mercaptobenzothiozole [14,39,41]. As per ICH M7 guideline, the cohort of concern (group of high potency mutagenic carcinogens) comprises aflatoxin-like, *N*-nitroso, and alkyl-azoxy compounds [31]. These structural groups are associated with high mutagenic potency; thus, intakes even below the TTC may pose a significant carcinogenic risk [31].

In 2021, PQRI published recommendations regarding E&L in parenteral drug products (PDP), including intravenous, subcutaneous, and intramuscular applications [40]. Given the predominantly aqueous nature of most PDP formulations, a SCT of 1.5 μg/day was proposed for individual organic leachables. However, a lower SCT may be warranted for certain compound classes such as those within the cohort of concern, i.e., aflatoxin-like substances, as well as *N*-nitroso and alkyl-azoxy compounds due to their higher toxicological risk [40]. The QT previously developed for OINDP was also evaluated for PDP. It was determined that for compounds that are neither mutagenic nor potentially mutagenic, a QT of 5 µg/day may be applied based on the endpoints of irritation and sensitization. If the total daily exposure to a leachable exceeds the threshold dose of 5 µg per day, the compound should be evaluated for its mutagenic potential, as well as for the potential to cause irritation or sensitization [40].

In December 2023, the FDA issued a Draft Guidance for Industry titled “Quality Considerations for Topical Ophthalmic Drug Products”, which outlines key quality attributes for ophthalmic drug products, such as solutions, suspensions, emulsions, gels, ointments, and creams, intended for topical application in and around the eye [42]. The guidance addresses E&L as part of the overall quality assessment. It supports the use of a safety threshold-based approach to evaluate the potential for E&L to leach into and/or interact with the drug product. The recommended leachable thresholds are expressed in parts per million (ppm—i.e., the parts of a leachable per unit mass of the ophthalmic drug product) and include

- Reporting threshold: 1 ppm
- Identification threshold: 10 ppm
- Qualification threshold: 20 ppm

Given the direct ocular application of ophthalmic drug products, the guidance emphasizes that any leachables exceeding the qualification threshold must undergo a safety assessment. The evaluation should address the ocular toxicity and irritancy potential, in addition to systemic safety, as appropriate. However, the tendency of the guideline is towards giving more importance to local toxicological effects [42]. Houston et al. have provided an overview of toxicological considerations for leachables in ophthalmic drug products [43].

Specifically for intravitreal exposure, there are currently no regulatory guidelines or applicable frameworks to establish TTC values for ocular toxicity. The criteria to be considered when establishing safety limits and permitted daily exposures (PDEs) for intravitreal route are well described within the paper by Rice et al. [44].

### 3.2. Translation of Toxicological Limits into Analytical Limits in Extractables and Leachables Analyses

SCT and QT are dose-based thresholds expressed in units of µg/day, which are highly relevant for toxicological risk assessment. However, these thresholds have limited direct applicability for analytical chemists, as they do not correspond to the concentration-based units (e.g., µg/mL) that are analytically measured. To bridge this gap, the dose-based thresholds must be converted into concentration-based analytical thresholds. In E&L analyses, such a threshold has been termed the AET, which is defined as the concentration (threshold) above which a leachable or extractable should be identified (i.e., its chemical structure elucidated), (semi)quantified, and reported to a toxicologist for safety assessment [5,39,40].

By definition, the AET establishes a threshold for the toxicological assessment of E&L. Chemical entities with concentrations exceeding the AET must be identified and quantified as a prerequisite prior to their toxicological evaluation since they may pose a safety risk. On the other hand, E&L present at concentrations equal to or below the AET are generally not subject to identification or quantification, as their potential to cause adverse effects is considered negligible [1,3,5]. It is crucial to understand that the AET is not a safety limit itself; it is a reporting threshold that ensures potentially concerning compounds are not overlooked.

As an illustrative example of AET calculation, we will consider a nasal spray drug product indicated for chronic (lifetime) use, with 100 labelled actuations per container, a maximum recommended patient exposure of 2 actuations per day, and a total fill volume of 10 mL. Under these conditions, the AET can be estimated, where SCT is the safety concern threshold for OINDP (0.15 μg/day as recommended in [14,39]) and V(MDD) is the volume of maximum daily dose:

(1) $$V(MDD)=\frac{10\frac{mL}{container}\times 2\frac{actuations}{day}}{100\frac{actuations}{container}}=0.20\;mL/day$$

(2) $$AET=\frac{SCT}{V(MDD)}=\frac{0.15\frac{\mu g}{day}}{0.20\frac{mL}{day}}=0.75\;µg/mL$$

Screening analyses of E&L require consideration of analytical uncertainty since the quantification of compounds is usually performed semi-quantitatively based on the internal or external standard(s), the response factor of which may significantly differ from the response factor of individual extractables and/or leachables. To account for that analytical variability, an uncertainty factor (UF) is applied to most AET calculations. Analytical uncertainty associated with a given analytical technique or method can be estimated by analysing a series of reference compounds to establish a response factor database. For OINDP, it is recommended that the estimated AET is lowered by a factor corresponding to 1% relative standard deviation (RSD) in an appropriately constituted response factor database, or by a factor of 50% of the estimated AET, whichever results in a greater AET reduction [14,39]. This conservative adjustment ensures that compounds with potentially significant toxicological relevance are not overlooked due to analytical variability. If the uncertainty factor of 50% is applied to the previous AET calculation, the final AET can be estimated as

(3) $$AET\left(final\right)=\frac{SCT}{V\left(MDD\right)}\times UF=\frac{0.15\frac{\mu g}{day}}{0.20\frac{mL}{day}}\times 0.5=0.375\;µg/mL$$

### 3.3. The Process of Toxicological Risk Assessment of Extractables and Leachables

**Figure 2:**
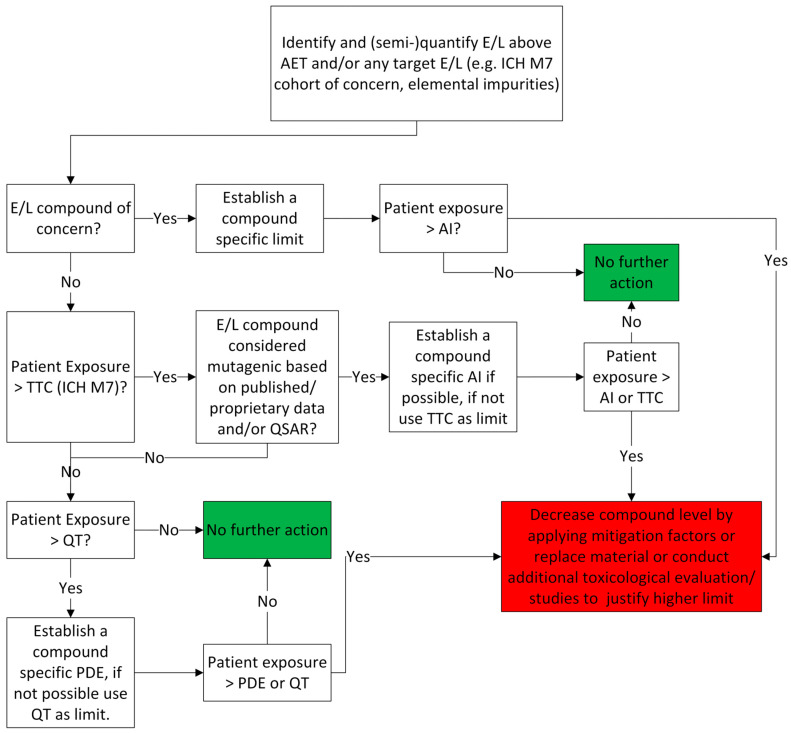
Example of an E&L safety evaluation process (adapted from [39,40,45]). More detailed overview of the E&L toxicological evaluation process and the determination of the applicable limits is presented in Figure 3. Abbreviations: Acceptable Intake (AI); Extractable/Leachable (E/L); Permitted Daily Exposure (PDE); Quantitative Structure–Activity Relationship (QSAR); Qualification Threshold (QT); Threshold of Toxicological Concern (TTC). Green colour: End of decision process, E&L levels within acceptable limits; Red colour: E&L levels above acceptable limits and further action needed.

The primary objective of toxicological risk assessment is to evaluate the potential impact of leachables on patient safety. Such assessments may also be performed for extractables, as they represent compounds that are probable leachables. A variety of approaches have been proposed for the toxicological evaluation of E&L [39,40,45], all following a similar general framework. An example of such a framework, often used in practice, is illustrated in Figure 2.

To initiate a toxicological risk assessment, appropriate input data must first be collected. For all relevant extractables and/or leachables detected at concentrations exceeding the AET, information on compound identity, such as IUPAC name, CAS number, chemical formula, and chemical structure, is required, along with the compound’s concentration in the sample/drug product. Ideally, both identity and concentration should be reported with an associated degree of certainty. If uncertainty exists, it must be appropriately accounted for in the risk assessment process.

The mechanics of toxicological risk assessment of E&L are defined in foundational guidance (ISO 10993-17:2023) and in the recently published draft ICH Q3E [46,47].

#### 3.3.1. Evaluation of Mutagenicity

The toxicological evaluation begins by determining the patient’s Estimated Daily Exposure (EDE) to the substance, which is calculated considering the DP maximum daily dose. Next, it needs to be determined if the extractable/leachable is a compound of concern, typically based on whether its chemical structure belongs to the ICH M7 cohort of concern [31]. If the extractable/leachable is identified as a compound of concern, a compound-specific acceptable intake (AI) must be established for it. This AI is then compared to the patient’s EDE. If the EDE exceeds this limit (EDE > AI), risk mitigation measures are required (as detailed below). This comparison is typically expressed as the Margin of Safety (MoS), calculated as the ratio between the AI and the patient’s EDE. A MoS value of less than 1 (i.e., AI < EDE) indicates a potential safety concern.

(4) $$MoS=\frac{Acceptable\;Exposure\;Level\;(e.g.,\;AI\;or\;PDE)}{Patient^{’}s\;EDE}$$

If the extractable/leachable is not a compound of concern, then in the next steps, the patient’s EDE is compared to the appropriate threshold for mutagenic impurities, the TTC limit from the ICH M7 guideline [31]. If the EDE is less than or equal to the TTC, the substance is considered to pose no safety concern from a mutagenicity point of view. However, the evaluation must still proceed to assess other toxicological endpoints (as outlined in Section 3.3.2 Evaluation of Other Toxicological Endpoints). Evaluation of other toxicological endpoints is required since in the case of LTL exposure duration, the toxicologically acceptable limit for mutagenic impurities might be higher than the acceptable limits for other toxicological endpoints (e.g., local irritation and sensitization, systemic toxicity).

Conversely, if the calculated patient exposure exceeds the TTC, the substance may pose a potential safety concern from mutagenicity point of view and should be further investigated. According to the ICH M7 guideline, mutagenic substances are “DNA reactive substances that have a potential to directly cause DNA damage when present at low levels leading to mutations and therefore, potentially causing cancer. This type of mutagenic carcinogen is usually detected in a bacterial reverse mutation (mutagenicity) assay” [31].

Firstly, the substance’s mutagenicity is evaluated by performing a literature overview of any available in vitro and in vivo experimental data evaluating the mutagenic potential of the substance. Negative experimental results from a reliable and well-performed bacterial mutagenicity assay provide sufficient evidence that a compound is not mutagenic.

In the absence of literature experimental data, or when its reliability and/or completeness are not satisfactory, the evaluation of mutagenicity may be complemented or entirely replaced with appropriate in silico tools. The ICH M7 guideline allows the usage of computational tools for evaluating the mutagenicity of various impurities. A computational toxicology assessment can be performed using quantitative structure-activity relationship (QSAR) methodologies that predict the outcome of a bacterial mutagenicity assay. Two complementary QSAR prediction approaches should be applied: expert rule-based and statistical-based. QSAR models utilizing these prediction methodologies should follow the general validation principles set forth by the Organization for Economic Co-operation and Development (OECD). The absence of structural alerts from two complementary QSAR methodologies (expert rule-based and statistical) is sufficient to conclude that the impurity is of no mutagenic concern, and no further testing is recommended. If warranted, the outcome of any computer system-based analysis can be reviewed with the use of expert knowledge in order to provide additional supportive evidence on the relevance of any positive, negative, conflicting, or inconclusive prediction and provide a rationale to support the final conclusion [31,48]. If the evaluation determines that the substance is mutagenic, the next step is to establish a substance-specific AI whenever possible [31,45]. If the AI cannot be established due to insufficient data, the patient’s EDE should be compared against the TTC, and the applicability of an LTL approach must be evaluated. When levels of the impurity cannot be controlled at an appropriate acceptable limit, it is recommended that the impurity is tested in an in vivo gene mutation assay to understand the relevance of the bacterial mutagenicity assay result under in vivo conditions. The selection of other in vivo genotoxicity assays should be scientifically justified based on knowledge of the substances‘ mechanism of action and expected target tissue exposure. Results in the appropriate in vivo assay may support setting compound-specific impurity limits [31]. Conversely, if the substance is not considered mutagenic, its safety assessment proceeds by evaluating other toxicological endpoints, as outlined in Section 3.3.2.

Substance-specific risk assessments to derive acceptable intakes should be applied instead of the TTC-based acceptable intakes where sufficient carcinogenicity data exist. For known mutagenic carcinogens, a substance-specific AI can be derived based on carcinogenic potency data, if available. The default approach involves linear extrapolation from the Tumorigenic Dose 50 (TD_50_) value, defined as the dose associated with a 50% tumour incidence (corresponding to a cancer risk probability of 1:2), as outlined in the ICH M7 guideline [31]. The substance-specific AI can be calculated using rodent carcinogenicity potency data, where the TD_50_ from the most sensitive species is divided by 50,000 to extrapolate to an acceptable lifetime cancer risk of 1 in 100,000. This methodology is analogous to that used for the derivation of the TTC. Furthermore, ICH M7 permits the derivation of class-specific AIs for impurities that are structurally related to known carcinogens, provided that a scientifically justified rationale for chemical similarity is presented along with supporting toxicological data [31]. Such a read-across approach is particularly useful in the assessment of E&L, where structural analogies among compounds are frequently observed. This approach is also gaining on its reliability, especially with the assistance of continuously evolving in silico tools that support the determination of the most appropriate surrogates with appropriate toxicological data available.

The TTC of 1.5 μg/day is considered protective for lifetime daily exposure; however, many drug products are administered less frequently or only for a limited duration of time. A fundamental principle in cancer risk assessment is the assumption that cancer risk correlates with cumulative dose. According to this concept, a continuous low-dose exposure over a lifetime is assumed to be associated with the same cancer risk as an equivalent cumulative exposure averaged over a shorter period of time—a relationship described by Haber’s Law. The TTC limit of 1.5 μg/day corresponds to a cumulative lifetime dose of 38.3 mg (1.5 μg/day × 25,550 days). Based on this principle, ICH M7 provides a series of adjusted limits, for individual and multiple mutagenic impurities, for shorter treatment durations, referred to as LTL limits (please refer to Table 4) [31,45]. The same approach may be followed for a substance-specific AI derived from the TD_50_ value.

If, after the additional evaluation, the levels of the mutagenic leachable cannot be toxicologically justified, appropriate risk mitigation measures must be implemented. These measures should aim to reduce the concentration of the mutagenic leachable in the drug product, for example, by replacing the packaging/manufacturing/administration component identified as the source of the impurity, pre-washing of components, or in the case of manufacturing components, pre-flushing and additional purification/isolation steps can be considered. Alternatively, another risk mitigation step may be to conduct additional toxicological evaluations or toxicological studies to determine if a higher, compound-specific AI can be justified.

If risk mitigation measures are not effectively implemented and fail to reduce the concentration of the mutagenic leachable to toxicologically acceptable levels, the drug product must be deemed unsafe and unsuitable for use.

#### 3.3.2. Evaluation of Other Toxicological Endpoints

If the leachable is considered to be non-mutagenic and does not raise SAR concerns, the next step is to compare its concentration to the QT of 5 µg/day. If the patient exposure is below or equal to this threshold, no further toxicological qualification is required. However, if the patient exposure exceeds 5 µg/day, additional toxicological evaluation is necessary to assess potential safety risks.

The safety assessment proceeds with the evaluation of other relevant toxicological endpoints that reflect potential risks to patient health, such as sensitization potential/local irritation, systemic toxicity (acute and repeated-dose), toxicokinetics, reproductive and developmental toxicity, and carcinogenicity [45]. At this point, the toxicological endpoints evaluated should be prioritized based on the specificities of the evaluated drug product, such as the route of administration, indication, and the patient target population. These considerations are well described within the ICH Q3E guideline with controls proportionate to the identified risk [47]. The toxicological evaluation should start with the assessment of local effects before systemic endpoints. The toxicological data that is considered should be obtained from reliable and well-performed studies that ideally are OECD- and good laboratory practice (GLP)-compliant.

Local safety is addressed prior to systemic endpoints because concentrations at the site of administration can exceed systemic levels, making local effects the potential risk driver for patient protection; for example, deposition in the lungs for inhalation products, contact with the ocular surface for ophthalmics, injection-site exposure for parenterals, and skin contact for dermal dosage forms. For topical ophthalmics, for example, the tendency of the FDA draft guideline is towards giving more importance to local toxicological effects [42], and less importance to systemic effects. The local assessment integrates literature evidence and read-across from structural analogues with relevant exposure conditions, New Approach Methodologies (NAMs) (including in silico alerts for protein reactivity/sensitization and irritation potential), and route-appropriate data when needed (e.g., focused nonclinical local-tolerance studies or human observational data). The hazard is evaluated based on estimated local concentration, derived from the measured leachable level in the product and route-specific delivery parameters (for example, nominal dose and deposition for inhalation; volume administered and contact time for ophthalmic use; bolus and tissue diffusion characteristics at the injection site for parenteral administration; application rate and area dose for dermal products). For these endpoints, experimental data from in vitro and in vivo eye and skin irritation studies and in vitro and in vivo respiratory and dermal sensitization studies are relevant and, for many substances, frequently available in the public domain. In light of the 3R principles (Replacement, Reduction, Refinement), in vivo studies are now largely replaced by in vitro studies using different models. For example, reconstructed human epidermis models have largely replaced traditional animal tests for skin irritation and corrosion and are increasingly used for skin sensitization [49].

It is, however, important to critically assess the study results with regard to their relevance for the product evaluated. In studies evaluating irritation potential, compounds at high concentrations are usually administered, which is a scenario very different from the low concentrations of E&L potentially present in the drug product. For instance, it is discussed by Masuda-Herrera et al. that for irritation potential, the concentration is the relevant parameter—when diluted, these same compounds are no longer considered irritants [50]. The United Nations (UN) considers hazard labelling for compounds that are corrosive to the eye or skin no longer necessary if they are below a concentration of 1% in the mixture. E&L are usually present at much lower concentrations, and it is highly unlikely that a low-level compound could result in an irritating effect when the concentration is several orders of magnitude below 1% and the pH of the drug product is within specification. Thus, any safety risk arising from the detected irritation potential may be considered less relevant if the exposure concentration is low.

A positive sensitization test also does not mean that a compound should by default be limited to the QT of 5 µg/day as proposed by PQRI for PDP [40] or that slightly higher exposures would by default represent a risk for patients. It is discussed in publications that the 5 µg/day limit is very conservative even for the most potent sensitizers (extreme and strong) (refer to Figure 6 and TABLE V within (Parris et al. [51])), as it has a disproportionately large safety margin to a human equivalent dose (see Table 5 within (Parris et al. [52])). Even for substances classified as strong or extreme skin sensitizers, the likelihood for induction of sensitization following parenteral exposure at 5 µg/day is considered negligible. As an illustrative example, authors describe the extreme skin sensitizer oxazolone, which has an estimated concentration 3 (EC3; the estimated concentration needed to produce a stimulation index of 3 in the murine local lymph node assay) value of 0.01%, resulting in a human equivalent dose of 416,667 µg/day, which is >83,000 fold above 5 µg. Strong sensitizers were determined to have at least an 833,333 safety margin to the 5 µg/day limit.

A substance is locally qualified when the weight of evidence supports no clinically relevant local irritation/sensitization at or above the maximum local concentration expected during use.

Once local risks are addressed, systemic hazards are considered, and a health-based limit is derived to judge patient exposure. For each endpoint, a combination of in vitro and/or in vivo studies may be employed to generate robust safety data. Broschard et al. provided a detailed summary of representative study types that may be employed in the safety assessment of E&L [45].

Systemic endpoints routinely considered are the following [46]:

General (repeated-dose) systemic toxicity. Subacute/subchronic/chronic studies (rodent and non-rodent) are screened to identify the most sensitive target organ and effect, with preference for the longest duration and most route-relevant dataset.

Reproductive and developmental toxicity. Fertility, embryo-foetal, and pre-/postnatal studies are reviewed, where the maternal or offspring no-effect levels (whichever is protective) inform on systemic limits for populations with reproductive potential or pregnancy exposure. In certain cases, potential embryo-foetal effects are not a clinically relevant risk for the intended patient population. For example, when a drug product’s administration is restricted to men and to women not of childbearing potential (post-menopausal or permanently sterilized) and is contraindicated in pregnancy.

Genotoxicity. The standard battery (Ames plus a cytogenetic/micronucleus assay) is interpreted within a risk-management framework; importantly, when a leachable is DNA-reactive (mutagenic), acceptable intakes are set under ICH M7(R2), as described in Section 3.3.1.

Carcinogenicity. If robust long-term data exist, non-genotoxic carcinogenic effects are handled by standard derivation of a safe limit (see further below); genotoxic carcinogens fall under ICH M7-based AIs (see Section 3.3.1).

Specific studies: immune, neuro, and endocrine-related systemic effects. Indicators arising within repeated-dose studies (e.g., organ weights/histopathology, neurobehavioral observations, hormone or immune endpoints) are considered; when specific studies are available, they are integrated as systemic hazards in the same derivation of a safe limit.

However, in the majority of cases, data completeness may not be satisfactory, the reliability of studies may be doubtful, or studies may not be available at all. In this case, besides the evaluation of mutagenicity, certain in silico tools may provide excellent support also to predict other toxicological endpoints, such as skin and respiratory sensitization, and eye and skin irritation. However, consensus remains that no harmonized/validated test guideline exists yet and there is no scientific consensus on how to measure external predictivity of in silico models [53]. OECD’s Detailed Review Paper is in public consultation, and recent reviews highlight the endpoint’s complexity and ongoing NAM development, so predictions should be treated within a weight-of-evidence framework and clearly caveated [54]. In vitro and in chemico assays can predict the skin sensitization potential, including those that are outside the applicability domain of existing non-animal assays.

Various in silico tools, such as Lhasa NEXUS and OECD QSAR Toolbox, continue to expand with automated workflows and frequent version updates; read-across should be mechanistically justified to strengthen dossiers by supporting a robust weight-of-evidence assessment of dermal sensitization while minimising new testing [55,56].

In cases when there are multiple toxicological endpoints identified for one chemical, the most sensitive systemic endpoint (the lowest dose with no effect observed in the most sensitive species) that leads to the lowest point of departure (POD) and the lowest derived safe limit should be selected as risk-determining. When multiple chemicals are observed that share the target organ or toxicological mechanism, a group safe limit may be established for these compounds, and cumulative dose addition and cumulative MoS are considered acceptable. When multiple chemicals are present with dissimilar modes of action, each of them should be evaluated independently as their cumulative action is unlikely and need not be aggregated unless mechanistic data suggest otherwise.

Once all available toxicological data have been gathered and critically assessed, the toxicologist determines whether the dataset is sufficiently robust to support the establishment of a PDE. To derive a compound-specific limit, such as a PDE, the toxicologist evaluates the data from all the available studies to identify the critical effect, defined as the most sensitive and/or relevant adverse effect in the non-clinical study. Ideally, a no observed effect level (NOEL) or no observed adverse effect level (NOAEL) should serve as the basis for deriving an exposure threshold. The NOEL or the lowest observed effect level (LOEL) for the identified critical effect should be used as the POD. From this POD, a PDE is calculated by applying appropriate modifying factors, as outlined in ISO 10993-17 and detailed in the ICH Q3C guideline [45,46,57].

According to the ICH Q3C guideline, a standard weight adjustment of 50 kg is proposed for the derivation of a PDE. The PDE is calculated based on the NOEL, or the LOEL from the most relevant animal study, using the following equation [57]:

(5) $$PDE=\frac{NOEL\times Weight\;Adjustment}{F1\times F2\times F3\times F4\times F5}$$

where the modifying factors are defined as follows (please refer to ICH Q3C for additional details [57]):

- F1: Accounts for extrapolation between species (takes into account the comparative surface area to body weight ratios for the species concerned and for humans).
- F2: Accounts for variability within the human population (intraspecies variability), protecting sensitive subpopulations. A default value of 10 is generally applied.
- F3: Accounts for the duration of the toxicity study when extrapolating from a subchronic study to a chronic exposure scenario. For example, a factor of 2 might be used to extrapolate from a 90-day toxicity study to a lifetime PDE.
- F4: Applied in cases of severe toxicity (e.g., non-genotoxic carcinogenicity, neurotoxicity, or teratogenicity).
- F5: Applied when a NOEL was not established and only LOEL is available—a factor of up to 10 may be used depending on the severity of the toxicity.

An additional modifying factor (F6) may be applied to account for route-to-route extrapolation as well as in cases when the target population includes sensitive subgroups, such as neonates [45].

Within the ICH Q3E, the term acceptable exposure (AE) level is used. According to ICH Q3E, the AE is based on the PDE methodology described in other ICH guidelines; however, AE is considered not necessarily to be the same as the PDE. Whereas the PDE is by definition an exposure level for lifetime and is applicable across many products, the product-specific AE takes into account the duration of exposure and maximum daily dose [47].

**Figure 3:**
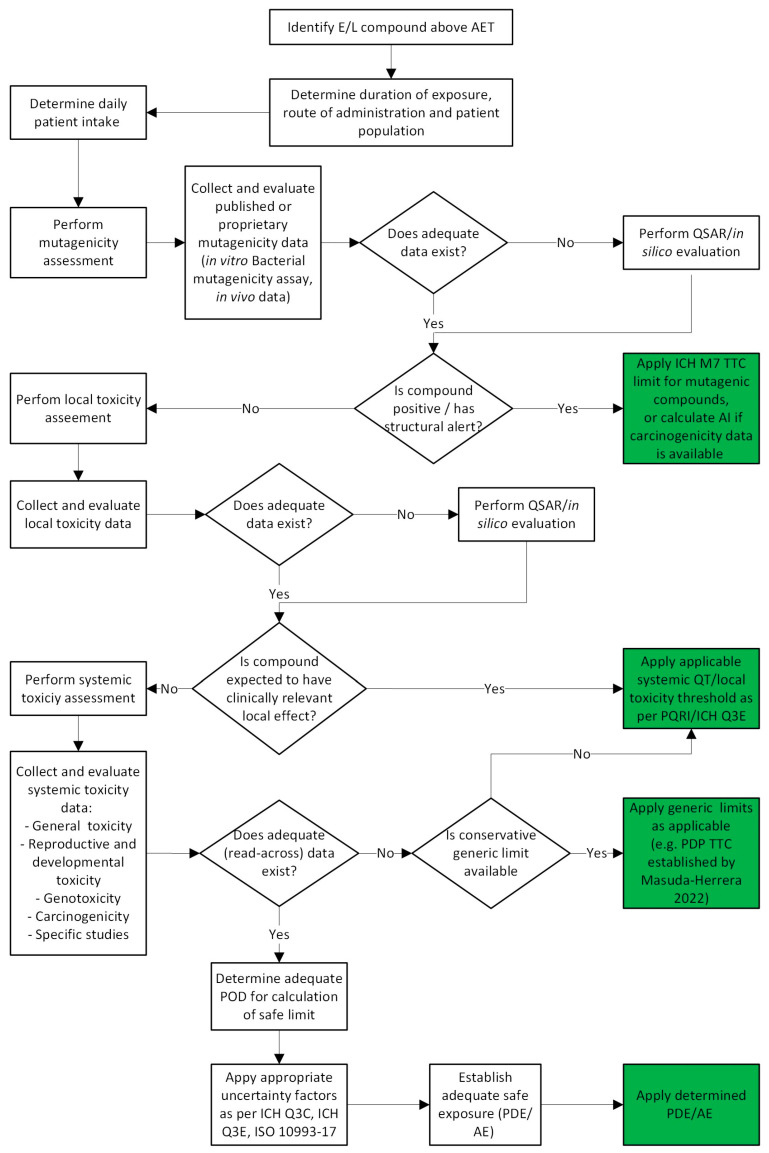
Workflow diagram for the toxicological evaluation process of E&L and determination of applicable limits. An example of general E&L safety evaluation process is presented in Figure 2. Abbreviations: Acceptable Intake (AI); Acceptable Exposure (AE); Extractable/Leachable (E/L); Permitted Daily Exposure (PDE); Parenteral Drug Product (PDP); Point of departure (POD); Product Quality Research Institute (PQRI); Quantitative Structure-Activity Relationship (QSAR); Qualification Threshold (QT); Threshold of Toxicological Concern (TTC). Masuda-Herrera 2022 is reference number [50]. Green colour: End of decision process, applicable limits have been identified or established.

The described workflow for the toxicological evaluation of E&L and the decision tree for the determination of applicable limits is summarized in Figure 3.

Parris et al. presented four case studies illustrating the derivation of PDE values for commonly encountered leachables: BPA, butylated hydroxytoluene (BHT), Irgafos^®^ 168, and Irganox^®^ 1010 [58].

If a PDE value can be established, the toxicological assessment proceeds by comparing the patient’s EDE to the PDE for a given extractable or leachable. This comparison is typically expressed as the MoS, calculated as the ratio between the PDE and the patient’s EDE (please refer to Equation (4)). A MoS value of less than 1 (i.e., PDE < EDE) indicates a potential safety concern.

In such cases, appropriate risk mitigation measures must be implemented with the aim of reducing the concentration of the leachable (as described above). If risk mitigation measures are not effectively implemented and fail to reduce the concentration of the leachable to toxicologically acceptable levels, the drug product must be deemed as unsafe and unsuitable for use. Conversely, if the MoS is equal to or greater than 1 (i.e., PDE ≥ patient exposure), the substance is considered to not pose a safety risk, and the toxicological assessment can be considered completed.

If a PDE cannot be established (in the absence of toxicological data), then a generic threshold may be applied instead (e.g., QT or modified Cramer) [5,45]. Significant efforts regarding this have recently been made by toxicological experts within The Extractables and Leachables Safety Information Exchange (ELSIE) Consortium, where a comprehensive review of their databases has been performed to develop duration-based non-mutagenic TTC, which is relevant for E&L in PDPs. The resulting parenteral TTC values are 35, 110, and 180 µg/day for human exposures of >10 years to lifetime, >1–10 years, and ≤1 year, respectively [50]. These parenteral TTCs are expected to be conservative for E&Ls that are considered non-mutagenic per ICH M7(R1) guidelines and have excluded or sufficiently justified as irrelevant their sensitizing/irritation potential [50]. In a more recent publication by this group, these proposed values were also evaluated to have high coverage also for the compounds that are predicted to have sensitizing potential, which is predominantly due to the fact that the generic limit for sensitizers of 5 µg/day was established in a very conservative way by applying significant safety factors [51].

Ultimately, the toxicological risk assessment yields one of two outcomes:

1. The drug product is qualified to be safe with respect to the presence of leachables (MoS value is more than 1).
2. The drug product is considered at risk due to the presence of leachables (MoS value is less than 1).

In the latter case, mitigation measures must be implemented to reduce the concentration of the leachable (as described above). If risk mitigation measures are not effectively implemented and fail to reduce the concentration of the leachable to toxicologically acceptable levels, the drug product is considered as unsafe and unsuitable for use.

Even when the MoS is more than 1, additional risk controls might still be required, particularly when the margin is minimal and cannot be appropriately justified. For example, a leachable substance approaching its safety threshold (e.g., patient’s EDE > 30% of PDE/AI) may warrant inclusion in material or DP specification for continued monitoring, to further confirm that patient safety is not compromised.

### 3.4. ICH Q3E Perspective on Toxicological Evaluation of Extractable and Leachables

The release of the draft ICH Q3E guideline “Guideline for Extractables and Leachables” represents an important milestone. Although the guideline is still in draft stage and subject to revision, it provides a direction in which the E&L field is likely to evolve in the future. The aim of the ICH Q3E guideline is to establish a globally harmonized, science-driven, and risk-based framework for the toxicological evaluation and control of E&L. The primary focus is on organic leachables. While the recommended methodologies for elemental analysis are within the scope of ICH Q3E, the safety assessment of elemental leachables (inorganic impurities) is outside its scope, as this is comprehensively addressed by ICH Q3D [17,47].

ICH Q3E represents the formal, global standardization of principles largely pioneered by PQRI. While fundamentally aligned in their risk-based, threshold-driven philosophies, ICH Q3E serves as a logical evolution, providing a harmonized guideline with a wider scope. Key refinements include the adoption of the SCT and QT concepts, which ICH Q3E harmonizes by linking their derivation directly to ICH M7 and new, data- and exposure duration-driven QTs. Critically, its applicability is expanded beyond the OINDP and Parenteral and Ophthalmic Drug Product (PODP) contexts. ICH Q3E thus elevates PQRI’s foundational work, creating a unified global standard that incorporates and refines two decades of scientific progress [47].

#### 3.4.1. Toxicological Thresholds

In ICH Q3E, the SCT is defined as a level of exposure below which a leachable is considered to present a negligible risk for both mutagenic and non-mutagenic toxic effects. It is important to note that the SCT is not applicable to a specific list of highly potent compounds, designated as “Class 1 leachables” within the guideline, which require a compound-specific evaluation regardless of their concentration. The SCT is not a single, universal value. Instead, it is a product-specific threshold that depends on both the route and duration of exposure (please refer to Table 5 and Table 6). For any given drug product, the SCT is established as the lowest of the three distinct toxicological thresholds: TTC and QT for systemic toxicity, and, if applicable, the threshold for local toxicity. By defining the SCT as the most conservative (i.e., lowest) of these values, the guideline ensures that the final threshold is protective against both the risk of mutagenicity and the risk of other systemic and local toxicities [47].

ICH Q3E adopts its TTC directly from the ICH M7 guideline, applying it for the assessment of systemic toxicity for oral, parenteral, dermal/transdermal, and inhalation routes of administration. This approach removes the specific, lower TTC for OINDP that was previously proposed by PQRI. As a direct consequence, the AET calculated in the previous example (Equation (3)) would be 10 times higher as per ICH Q3E (Equation (6)). Interestingly, the same SCT of 1.5 µg/day for OINDP was also proposed in the draft revision of USP <1664.1>, suggesting its potential adoption as a standard regulatory practice [59]. The final AET calculation, based on an SCT of 1.5 μg/day for OINDP as recommended by ICH Q3E and USP <1664.1>, is presented below [47,59]:

(6) $$AET\left(final\right)=\frac{SCT}{V\left(MDD\right)}\times UF=\frac{1.5\frac{\mu g}{day}}{0.20\frac{mL}{day}}\times 0.5=3.75\;µg/mL$$

The QT within the Q3E framework serves as the trigger point for conducting an in-depth, compound-specific toxicological assessment for non-mutagenic endpoints. A significant advancement in the Q3E guideline is that the QT values are not arbitrary or based on a single conservative assumption. Oral and parenteral QT values for systemic toxicity were derived from a comprehensive and robust scientific review of the PDEs for approximately 330 compounds that are potential leachables (please refer to Table 5). While systemic toxicity QT values for inhalation, dermal and transdermal routes have been established based on parenteral QT in lieu of available PDE values. Crucially, the QT is not a single value but is tailored to the specific context of the drug product, varying based on the route of administration and the duration of patient exposure. This introduces a level of scientific refinement that was absent from many previous, non-harmonized approaches. For example, the guideline establishes different QT values for chronic exposure via different routes, such as 48 µg/day for oral products compared to a more stringent 12 µg/day for parenteral products. This difference is due to higher toxicological risk associated with parenteral administration, which results in 100% systemic bioavailability and bypasses the body’s natural metabolic and detoxification defences in the gastrointestinal tract and liver. This directly links the clinical context of use to the required depth and rigor of the scientific safety investigation [47]. Moreover, the guideline establishes a higher QT in the case of short-term exposure.

In addition to systemic, local toxicity thresholds for leachables in topical ophthalmic, subcutaneous/intradermal, dermal/transdermal and inhalation drug products are also presented in the ICH Q3E guideline (please refer to Table 6).

For other routes of administration, the concepts defined in the ICH Q3E may be followed to determine the acceptable toxicity threshold [47].

#### 3.4.2. Classification of Leachables

When analytical testing reveals the presence of a leachable at a concentration that results in a patient’s EDE exceeding the established SCT, a formal toxicological qualification process is required. The ICH Q3E guideline provides a structured and systematic framework for this process, which involves classifying the leachable based on its hazard potential and, if necessary, deriving a compound-specific toxicological limit. This process integrates established principles of toxicological risk assessment with novel approaches tailored specifically to the challenges of E&L evaluation, including the formalization of route-to-route extrapolation and the encouragement of modern toxicological methods [47,57].

To facilitate a risk-based approach and prioritize assessment efforts, ICH Q3E introduces a three-tiered classification system for leachables. This system acts as an initial triage, allowing toxicologists to quickly categorize an identified leachable based on its known or predicted hazard profile and determine the appropriate path forward [47].

- Class 1 (Leachables to be avoided): This category is reserved for compounds of high concern for which the default safety thresholds are not considered sufficiently protective. These are substances that should be avoided in materials used for pharmaceutical manufacturing and packaging whenever feasible.
  This class includes
  - Highly potent mutagenic carcinogens, such as those belonging to the ICH M7 cohort of concern (i.e., *N*-nitroso-, aflatoxin-like-, and alkyl-azoxy compounds).
  - ICH M7 Class 1 compounds with an AI below the standard TTC threshold of 1.5 µg/day.
  - Certain highly potent non-mutagenic compounds for which the established QT values may not be protective of patient safety, such as BPA and benzo[a]pyrene.

For any Class 1 leachable, exposure must be controlled to a compound-specific acceptable limit, which is often significantly lower than the default thresholds [47].

- Class 2 (Leachables to be limited): This serves as the default category for the vast majority of identified leachables that do not meet the criteria for Class 1 or Class 3. If a Class 2 leachable is present at an exposure level exceeding the applicable SCT (QT for non-mutagenic systemic/local toxicity or TTC for mutagenic potential), it requires a compound-specific toxicological risk assessment to establish a safe level of exposure for the specific drug product [47].
- Class 3 (Leachables with relatively low toxic potential): This category includes compounds that are known to have a low toxicity potential. These are substances for which a robust toxicological database exists and a chronic PDE has been established to be well above the levels at which leachables are typically observed (specifically, a PDE greater than or equal to 1 mg/day). These compounds would not require further safety qualification if observed at daily exposure levels below 1.0 mg/day [47].

This pragmatic approach will reduce redundant toxicological work for common, well-understood, low-risk substances. Most of the Class 3 compounds are very common extractables from polymeric materials. Without the Class 3 designation, every company identifying, e.g., antioxidant BHT above the QT would be required to conduct its own independent literature review and PDE derivation. By creating this category and publishing official supporting monographs with pre-calculated PDEs, ICH is effectively pre-qualifying these substances, creating a significant efficiency gain for the industry by preventing countless toxicologists from repeatedly performing the exact same assessment on the same well-studied compound [47].

#### 3.4.3. The Toxicological Evaluation Process for E&L

The toxicological evaluation process as per ICH Q3E is very similar to the one described above in Section 3.3 (The Process of Toxicological Risk Assessment of Extractables and Leachables) and proceeds as follows [47]:

1. Organic leachables exceeding AET based on SCT or any target leachables (e.g., Class 1) should be identified, quantified, and reported for toxicological evaluation.
  - In case toxicologically justified, tentative structure identification can also be considered sufficient in certain cases.
  - If a leachable is an elemental impurity, evaluation should be performed in line with ICH Q3D.
2. For Class 2 leachables that exceed the relevant safety threshold (TTC or QT), for Class 3 leachables exceeding the levels of 1.0 mg/day, and for Class 1 leachables, the core of the toxicological qualification process is the derivation of a compound-specific AI or PDE.
  - In the case of target analyses of Class 1 leachables, their AI should be derived as the initial step in the process, before the start of analysis, to inform the analytical chemists about the required limit of detection and limit of quantification.
  - When no adequate toxicological data is available for a given leachable to conduct a safety risk assessment, a read-across approach may be used if appropriately justified.
  - ICH Q3E recommends establishing the MoS [47].
3. If the concentration of a leachable is below the relevant safety threshold (TTC or QT or AI or PDE), no further action is required.
  - In line with ICH Q3A and ICH Q3B, the ICH Q3E recommends that genotoxicity studies are considered if daily exposure to a leachable is >1 mg/day [38,47,60].
4. If the concentration of a leachable is above the relevant safety threshold (TTC or QT or AI or PDE) and it cannot be reduced, further toxicological risk assessment can be performed considering patient population, duration of use, and route of exposure. Generation of additional toxicological data should be considered as well.
  - If further risk assessment supports safety, then no further action is required.
  - If further risk assessment does not support the safety, then an appropriate risk mitigation strategy should be employed. In the end, the adequacy of the risk mitigation strategy has to be verified via the leachable study [47].

The methodology for deriving a PDE is consistent with well-established principles used in other ICH guidelines (e.g., Q3C for residual solvents) and by regulatory bodies worldwide, but Q3E recommends introducing a specific modifications for leachables such as modifying factors F6 for differences in bioavailability when route-to-route extrapolation is required and F7 for uncertainty associated with a read-across approach, which can be applied when using toxicological data from a surrogate compound to assess the target leachable. The value of F7 depends on the scientific robustness of the read-across justification, which is typically based on structural similarity, shared physicochemical properties, and common metabolic pathways between the surrogate and target compounds [47].

The ICH Q3E provides guidance to establish acceptable exposure levels for leachables that may exceed the calculated PDE under specific circumstances, provided that product-specific factors are considered. Such situations include intermittent or short-term administration (≤30 days), treatment of limited patient populations, or use in critical indications such as life-threatening conditions and rare diseases [47]. For drugs administered for less than a lifetime, a lower F3 factor may be applied when short-term toxicity data serve as the POD, resulting in an acceptable exposure level rather than a PDE [47].

#### 3.4.4. Embracing Modern Toxicology

In alignment with global trends to reduce, refine, and replace animal testing (the “3Rs”), the ICH Q3E guideline encourages the use of NAMs to support safety evaluations. NAMs encompass a range of modern toxicological tools, including the following:

- In silico (computational) toxicology: Using computer models to predict a chemical’s toxicological properties based on its structure. This is widely accepted for assessing mutagenicity potential (per ICH M7) and is also encouraged for endpoints like skin sensitization and irritation [31,47].
- In vitro and in chemico assays: Using cell-based or chemical reactivity assays to assess specific toxicological endpoints, such as dermal sensitization.
- Read-across strategies: As formalized by the F7 factor, this involves using data from well-studied chemicals to assess the toxicity of structurally similar, data-poor chemicals.

This formal endorsement signals clear regulatory acceptance of these modern tools. For many data-poor leachables, NAMs provide an efficient and scientifically powerful means of assessing hazard without requiring new, time-consuming, and costly animal studies. This allows for a more agile and mechanistically informed approach to toxicological risk assessment, focusing resources on the leachables of greatest potential concern [47].

## 4. Emerging Challenges

Despite the significant progress made in standardizing the E&L assessment framework, the field continues to face new and evolving challenges that demand ongoing scientific and regulatory innovation.

The rapid growth of biopharmaceuticals, including cell and gene therapies, presents unique E&L challenges. These products are often highly complex and can be highly sensitive to the presence of leachables, which may impact cell viability, protein stability, or immunogenicity in ways not seen with traditional small-molecule drugs [61]. The complex formulations used for these products can also possess unique leaching characteristics, requiring tailored assessment strategies.

This trend has converged with the industry-wide shift from fixed stainless-steel equipment to disposable single-use systems (SUS). While SUS (e.g., plastic bioreactor bags, tubing, filters) offer major advantages in flexibility, cost, and sterilization, they have introduced a new landscape of polymeric materials and E&L risks [26,27]. A notable example is bDtBPP, which has been shown to exhibit cytotoxic effects at concentrations well below the ppm range, significantly reducing cell growth and thereby reducing drug substance yields [26,27]. To address these risks, USP has introduced chapter USP <665>. This standard, becoming official in May 2026, establishes requirements for the characterization and qualification of plastic components/systems used for production of pharmaceutical drug products and biopharmaceutical drug substances and drug products [2,62].

Furthermore, the relentless pace of innovation in material science continuously introduces new polymers, additives, and composites into the supply chain. Each new material requires a thorough E&L evaluation to establish its safety profile. Advanced manufacturing technologies, such as 3D printing, create novel material-process combinations that may have unique E&L profiles requiring dedicated investigation. Regulatory agencies have begun to address this area; for example, the FDA’s guidance “Technical Considerations for Additive Manufactured Medical Devices” outlines critical validation parameters that are increasingly relevant for establishing the safety of pharmaceutical components produced via these advanced methods [63].

Sustainability initiatives, like the recycling of polymers, also pose new E&L questions that must be addressed. A notable example is the European Union (EU) Packaging and Packaging Waste Regulation (PPWR), Regulation (EU) 2025/40, which entered into force on 11 February 2025 and will apply fully from 12 August 2026 [64]. This regulation establishes requirements for packaging design, reuse systems, and waste management, including mandatory recyclability and minimum recycled content for most packaging by 2030 [64]. However, exemptions exist for healthcare-related packaging. At present, immediate packaging in direct contact with medicinal products, outer packaging necessary to maintain product quality, and contact-sensitive plastic packaging for medical devices and in vitro diagnostics are not subject to recyclability or recycled content [64].

Regulatory developments add another layer of complexity. The proposed ban on per- and polyfluoroalkyl substances (PFAS) submitted to European Chemicals Agency (ECHA) under the Registration, Evaluation, Authorisation and Restriction of Chemicals (REACH) regulation, for example, presents a paradox from an E&L point of view [65,66]. While the ban aims to protect public health and the environment by eliminating the use of PFAS compounds, these substances are also critical for producing highly inert components (e.g., polytetrafluoroethylene (PTFE) tubing) and effective protective barriers (e.g., PTFE-coated rubber stoppers). The removal of these proven materials and coatings, without suitable and readily available alternatives, could inadvertently increase the leaching of other substances, thus creating new E&L challenges. While the EU pursues potential restrictions of PFAS, the United States is currently in a phase of data collection and supply chain transparency, codified by the Toxic Substances Control Act (TSCA) Section 8(a)(7) [67].

Furthermore, existing toxicological frameworks are expected to continuously evolve. For instance, QTs proposed within ICH Q3E will likely be further refined as PDE values are derived for more compounds. Simultaneously, safety information gaps persist for certain high-risk routes of administration (e.g., intravitreal, intracerebral, intrathecal, and epidural), which require additional data to establish specific safety limits. Addressing these gaps highlights the importance of collaborative, cross-industry initiatives such as the ELSIE and PQRI consortia.

The availability of toxicological data is currently marked by a substantial disparity between the volume of information generated within industry and the proportion that is accessible to the public domain. While regulatory authorities routinely receive and evaluate extensive proprietary datasets, available data is restricted to publicly available REACH registration dossiers and the peer-reviewed literature. These sources frequently provide incomplete study descriptions and limited access to underlying datasets, restricting independent scientific evaluation. Bridging this gap will necessitate increased collaboration across regulatory agencies, industry, and academia, alongside the development of practical frameworks for data sharing, anonymization, and systematic aggregation of historical industry data. Enhancing transparency and promoting collective responsibility for toxicological data curation would improve the scientific robustness of hazard identification, minimize redundant animal testing, and ultimately support more efficient and reliable chemical risk assessment. Notably, cross-industry initiatives such as the ELSIE and PQRI consortia demonstrate the value of collaborative approaches, particularly through joint experimental data gathering, compound categorization, and the derivation of PDEs based on proprietary datasets. Such efforts enable the establishment of scientifically justified exposure limits for substances with limited, unreliable, or absent publicly available toxicological data, thereby reducing the need for the generation of new toxicological data. Examples include the previously described generic duration-based TTCs, which were developed by the ELSIE consortium members and are applicable to non-mutagenic E&Ls in PDPs. It is worth mentioning the development of QSAR models that will offer further practical means to support safety assessment of E&L. These tools will enable structure-based prediction of toxicological properties and can be applied within a weight-of-evidence framework to address data gaps without additional animal testing. Importantly, ongoing methodological advances, expanding training datasets with data donations, and improved model validation are steadily increasing the reliability and regulatory relevance of in silico tools, further strengthening their role in safety evaluation in data-poor scenarios.

Regulatory authorities, including the European Medicines Agency (EMA), and the FDA outline concrete opportunities and propose practical pathways to minimize or replace the use of laboratory animals across multiple regulatory contexts [68,69]. Evaluation and qualification of leachables and extractables should be no exception. Within this evolving framework, ICH Q3E represents a significant milestone, as it is the first guideline within the ICH Quality portfolio to explicitly incorporate NAMs into a globally harmonized and legally binding regulatory standard. In contrast to earlier Q3 guidelines, which relied predominantly on experimental testing, Q3E formally recognizes alternative, non-animal approaches grounded in mechanistic understanding, computational modelling, and weight-of-evidence evaluation. Notably, the guideline explicitly promotes the application of in silico toxicology and read-across methodologies for the safety assessment of leachables, with the clear objective of replacing animal testing wherever scientifically justified.

Ultimately, the quality of a toxicological assessment is only as good as the analytical data it relies upon, highlighting the deep interconnection between these two fields. It is critical to detect and identify all E&L compounds present above AET with appropriate certainty. If uncertainty exists, it must be formally accounted for in both the analytical and toxicological assessments. This may present a significant challenge for drug products with a very low AET, such as large-volume parenterals (LVPs), where current analytical detection capabilities might face significant hurdles in achieving the required sensitivity. Therefore, close and continuous collaboration between analytical chemists and toxicologists is and will remain critical to ensure robust E&L quality risk management.

## 5. Conclusions

This review presents a structured workflow for the toxicological risk assessment of E&L, supported by illustrative case studies that highlight a long history of pharmaceutical product quality and safety being compromised due to interactions with leachables originating from manufacturing components, packaging systems, and administration materials. These findings underscore the critical importance of implementing practical risk-based strategies for comprehensive E&L characterization, coupled with rigorous toxicological assessment, as an integral part of pharmaceutical development. Such evaluations are essential to ensure the quality, safety, efficacy, and stability of drug products throughout their lifecycle.

Looking ahead, the implementation of ICH Q3E will mark a new era of global regulatory harmonization, demanding a holistic and knowledge-based approach to E&L control throughout the product lifecycle. Continued advancements in analytical instrumentation and in silico toxicology will further enhance the ability to identify and assess risks with greater precision and efficiency. While challenges related to unknown compounds, complex mixtures, and novel therapies will persist, the fundamental principles of science-driven risk assessment and a continuous prioritization of patient safety will continue to guide the field forward. Ultimately, a robust and well-executed E&L program is not merely a regulatory requirement; it is a manufacturer’s commitment to the efficacy, quality, and safety of their products.

## Figures and Tables

**Table 2:** Risk-based approach to consideration of leachables (adapted from [3,36]).

Degree of Concern Associated with the Route of Administration	Likelihood of Packaging Component-Dosage Form Interaction		
	High	Medium	Low
Highest	Inhalation aerosols and sprays	Injections and injectable suspensions Inhalation solutions	Sterile powders and powders for injection Inhalation powders
High	Transdermal ointments and patches	Ophthalmic solutions and suspensions Nasal aerosols and sprays	/
Low	Topical solutions and suspensions Topical and lingual aerosols Oral solutions and suspensions	/	Oral tablets and oral (hard and soft gelatine) capsules Topical powders; oral powders

/—no example of dosage form defined.

**Table 3:** Overview of current toxicological limits used in the E&L analyses (refer to Tables 5 and 6 for future directions).

Route of Administration	Threshold Type	Recommended Limit	Reference Guideline
OINDP	SCT	0.15 µg/day ^1^	PQRI, USP <1664.1> [14,39]
	QT	5 µg/day	
PDP	SCT	1.5 µg/day ^2^	PQRI [40]
	QT	5 µg/day	
Topical ophthalmic	Reporting Threshold	1 ppm	FDA (Draft guidance) [42]
	Identification threshold	10 ppm	
	Qualification threshold	20 ppm	

^1^ Lower limits may apply for “special case” compounds (PAH, *N*-nitrosamines and 2-mercaptobenzothiozole) [14,39]. ^2^ Lower limits may apply for cohort of concern compounds (aflatoxin-like, *N*-nitroso, and alkyl-azoxy compounds) [40]. Abbreviations: Food and Drug Administration (FDA); Orally inhaled and nasal drug products (OINDP); Parenteral drug product (PDP); Product Quality Research Institute (PQRI); Qualification threshold (QT); Safety concern threshold (SCT); United States Pharmacopeia (USP).

**Table 4:** Acceptable daily intakes for individual and multiple mutagenic impurities based on the duration of treatment (adapted from [31]).

Duration of Treatment	≤1 Month	>1–12 Months	>1–10 Years	>10 Years to Lifetime
Acceptable daily intake for individual mutagenic impurity [µg/day]	120	20	10	1.5
Acceptable total daily intakes for multiple mutagenic impurities [µg/day]	120	60	30	5

**Table 5:** ICH Q3E systemic toxicity thresholds (adapted from [47]).

Systemic Toxicity Thresholds				
Exposure Duration	Oral		Parenteral, Dermal/Transdermal, Inhalation	
	TTC	QT	TTC	QT
>10 years	1.5 μg/day	48 μg/day	1.5 μg/day	12 μg/day
>1 to 10 Years	10 μg/day		10 μg/day	
>1 Month to 1 Year	20 μg/day		20 μg/day	
≤1 Month	120 μg/day	136 μg/day	120 μg/day	26 μg/day

Abbreviations: Qualification Threshold (QT); Threshold of Toxicological Concern (TTC).

**Table 6:** ICH Q3E local toxicity thresholds (adapted from [47]).

Local Toxicity Thresholds				
Topical Ophthalmic	Subcutaneous and Intradermal	Dermal and Transdermal	Intracerebral, Intrathecal, Epidural and Intraocular	Inhalation
20 ppm	50 ppm	500 ppm	Compound-specific evaluation	5 µg/day

## Data Availability

No new data were created or analyzed in this study. Data sharing is not applicable to this article.

## Abbreviations

The following abbreviations are used in this manuscript:

3R	Replacement, Reduction, Refinement
AE	Acceptable exposure
AET	Analytical Evaluation Threshold
AI	Acceptable intake
bDtBPP	*bis*(2,4-di-*tert*-butylphenyl)phosphate
BHT	Butylated hydroxytoluene
BPA	Bisphenol A
CHO	Chinese Hamster Ovary
DEHP	Di(2-ethylhexyl) phthalate
DNA	deoxyribonucleic acid
DP	Drug product
DS	Drug substance
EC3	Estimated concentration 3
ECHA	European Chemicals Agency
EDE	Estimated daily exposure
ELSIE	Extractables and Leachables Safety Information Exchange
EMA	European Medicines Agency
EPO	Erythropoietin
EU	European Union
E&L	Extractables and leachables
FDA	Food and Drug Administration
GLP	Good laboratory practice
IgG	Immunoglobulin G
IUPAC	International union of pure and applied chemistry
LOD	Limit of Detection
LOEL	Lowest observed effect level
LOQ	Limit of Quantitation
LTL	Less-than-lifetime
LVP	Large-volume parenterals
MBT	2-Mercapto-benzothiazole
MoS	Margin of safety
NAMs	New Approach Methodologies
NDBA	*N*-Nitrosodibutylamine
NDMA	*N*-Nitrosodimethylamine
NOAEL	No observed adverse effect level
NOEL	No observed effect level
OECD	Organization for Economic Co-operation and Development
OINDP	Orally inhaled and nasal drug products
PAHs	Polycyclic aromatic hydrocarbons
PDE	Permitted daily exposure
PDP	Parenteral drug products
PFAS	Per- and polyfluoroalkyl substances
PNAs	Polynuclear aromatic hydrocarbons
POD	Point of departure
PODP	Parenteral and Ophthalmic Drug Product
ppm	Parts per million
PPWR	Packaging and Packaging Waste Regulation
PQRI	Product Quality Research Institute
PRCA	Pure red cell aplasia
PTFE	Polytetrafluoroethylene
PVC	Polyvinyl chloride
REACH	Registration, Evaluation, Authorisation and Restriction of Chemicals
QSAR	Quantitative structure-activity relationship
QT	Qualification threshold
RSD	Relative standard deviation
SAR	Structure–activity relationship
SCT	Safety concern threshold
TD_50_	Tumorigenic dose 50
TSCA	Toxic Substances Control Act
TTC	Threshold of toxicological concern
UN	United Nations
USP	United States Pharmacopeia
UV	Ultraviolet
V(MDD)	Volume of maximum daily dose
